# Epithelial-Mesenchymal Transition Indexes in Triple-Negative Breast Cancer Progression and Metastases

**DOI:** 10.7759/cureus.68761

**Published:** 2024-09-06

**Authors:** Shota Kepuladze, George Burkadze, Irakli Kokhreidze

**Affiliations:** 1 Pathology and Oncology, Tbilisi State Medical University, Tbilisi, GEO; 2 Molecular Pathology, Tbilisi State Medical University, Tbilisi, GEO; 3 Oncology, Tbilisi State Medical University, Tbilisi, GEO

**Keywords:** emt index, epithelial-mesenchymal transition, tils, triple-negative breast cancer, tumor buds

## Abstract

Background

Triple-negative breast cancer (TNBC) is a highly aggressive subtype of breast cancer characterized by the lack of expression of estrogen and progesterone receptors and the absence of HER2 protein overexpression or gene amplification. How TNBC becomes so aggressive at the molecular level is not yet fully understood. The epithelial-mesenchymal transition (EMT) has been increasingly recognized as playing a pivotal role in cancer progression and metastasis. This study aimed to elucidate the connection between TNBC progression with EMT-related markers, including vimentin, beta-catenin, and E-cadherin.

Methodology

Rigorous immunohistochemical analysis was employed to assess the expression of vimentin, beta-catenin, and E-cadherin in primary tumors, tumor buds, and lymph node metastases (LNMs) from 137 cases with an invasive ductal carcinoma triple-negative phenotype diagnosed between 2018 and 2024. The EMT index, which was especially important in our work, is the sum of vimentin and beta-catenin expression divided by that of E-cadherin. Estimated Pearson correlation, multiple linear regression, and Kruskal-Wallis tests were used to determine the relationships of the EMT index with tumor buds and tumor-infiltrating lymphocytes (TILs).

Results

Vimentin highly correlated within separate regions of interest with Pearson correlation ranging from 0.90 to 0.92 (p < 0.001). Strong negative correlations between E-cadherin and vimentin (r = −0.81 to - 0.89, p < 0.001) showed its role in preserving the epithelial phenotype. The presence of tumor buds, aggregates, or clusters of cancer cells shed from the primary tumor mass invading the connective tissue showed very strong associations with the EMT index (r = 0.91, p < 0.001). Its presence is suggestive of aggressive disease and may identify a high‐risk subpopulation that may benefit from more active surveillance or adjuvant treatment. Similarly, TILs correlated inversely with the EMT index (r = -0.90, p < 0.001). The most significant predictor of the EMT index, i.e., vimentin, had a model R-squared value of 1.000 in the regression analysis.

Conclusions

This study brings to light the importance of EMT-related markers in TNBC progression, with special emphasis on tumor buds as possible prognostic indicators for aggressive disease. The negative correlation of TILs with the EMT index indicates that an effective immune response could antagonize EMT-mediated tumor progression. These results suggest that EMT-based treatments in TNBC should be designed from a multimarker perspective by including interactions among several markers to optimize predictions and therapeutics. The results hold the potential to set future research directions and actionable outcomes that could influence clinical utility in the battle against TNBC.

## Introduction

Breast cancer is one example of a very variable disease with different molecular subtypes, each representing specific clinical and biological features. Triple-negative breast cancer (TNBC) is a highly aggressive subtype of breast cancer characterized by the lack of expression of estrogen and progesterone receptors and the absence of HER2 protein overexpression or gene amplification [1,2].

The molecular basis that underlies the aggressive nature of TNBC remains an enigma. Mounting evidence now indicates that epithelial-mesenchymal transition (EMT) may be important in the evolution and dissemination of this subgroup [3]. However, the dissemination of cancer cells to remote organs is closely linked with the acquisition by tumor cells of attributes associated with a process known as EMT, which allows them to invade surrounding tissues and reach blood vessels that are close so they can travel long distances before seeding at distant sites [4,5]. The highly coordinated and invasive process is operated by an order of events that consists mainly of the invasion and intravasation of primary tumor cells into blood circulation flowing through the stroma [4-7].

Expressions of panel epithelial and mesenchymal markers (e.g., E-cadherin, vimentin, β-catenin) have served as surrogates for the assessment degree of TNBC plasticity [4,7]. These markers can offer important clues regarding the metastatic potential of the disease and may inform possible targeted therapies.

In this study, we examined the relationship between the expression of vimentin, β-catenin, or E-cadherin and clinicopathological features in human TNBCs [7,8]. The study will specifically analyze these markers by immunohistochemistry in primary tumors and tumor buds, which are clusters of cells detached from the primary cancer found at invasive margins predicted to be a sign of EMT. These results will aid in discovering the molecular basis for the aggressiveness of TNBC. These might provide clues for future, better-targeted therapies.

## Materials and methods

The Ethics Commission of Tbilisi State Medical University approved the study (approval number: 08.10.2020). The guidelines provided by the College of American Pathologists were followed during sample collection and processing.

Two pathologists independently performed case diagnosis and immunohistochemical evaluation (VS, GB). The data was analyzed using SPSS version 26 software (IBM Corp., Armonk, NY, USA).

The results of the expression of the immunohistochemical markers in the study represent the percentage. To calculate the EMT index, the sum of vimentin and β-catenin expression percentages was compared to the percentage of E-cadherin expression. Two indicators were used, namely, the EMT index, which measures epithelial-mesenchymal transformation in the tumor’s main focus, and the EMT index TB, which measures epithelial-mesenchymal transformation based on the expression of markers in defined tumor buds. The study aimed to determine the EMT index in triple-negative tumors of different stages, both in the main focus and in tumor buds in lymph node-positive and node-negative cases.

This retrograde cohort study used formalin-fixed and paraffin-embedded blocks of cases diagnosed in 2018-2024 at the Tbilisi State Medical University Educational, Scientific, and Diagnostic Laboratory. Inclusion criteria for the study were untreated (without neoadjuvant therapy) postoperative cases of invasive ductal carcinoma of the breast with triple-negative phenotype performed by lymph dissection.

**Figure 1:**
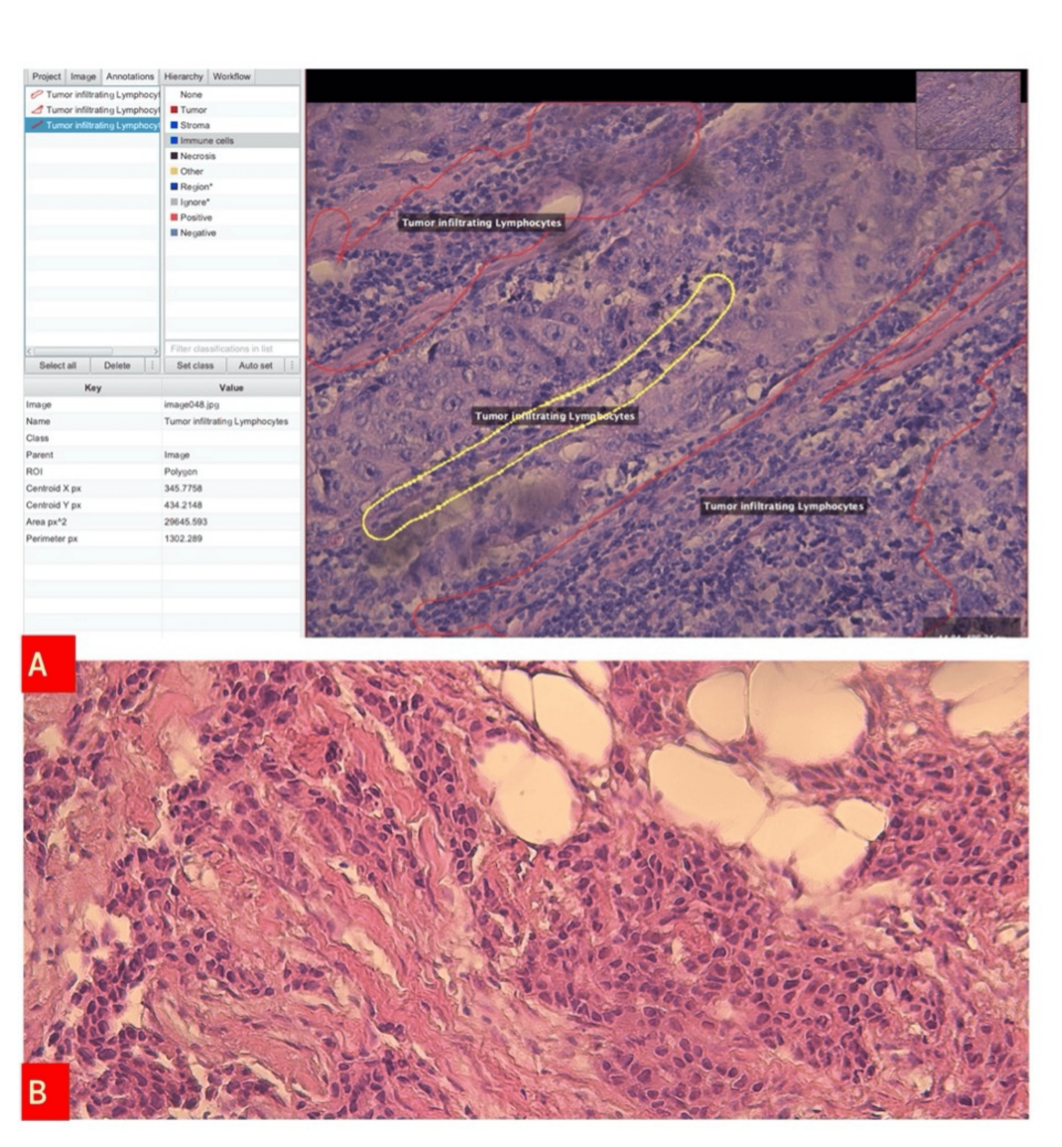
(A) Tumor-infiltrating lymphocytes were evaluated as 70% Qupath Program image (hematoxylin and eosin (H&E) 200×. (B) Tumor-infiltrating lymphocytes, <5% (H&E 200×).

The information collected for the research algorithm included a combination of various parameters, such as the pathological TNM classification of the tumor, the presence of lymphovascular invasion, and the degree of tumor differentiation. Evaluation of the tumor buds was performed using a quantitative method, which involves determining the number of cancer cellular clusters consisting of fewer than five cells in the maximum invasive field of the selected tumor (the so-called hot spot) in the high magnification field of view (400× magnification) [9,10]. The number of tumor buds in the survey data represented the total invasive front counts (Figure 1).

The percentage of tumor-infiltrating lymphocytes (TILs) was also evaluated according to the recommendations provided by the International TILs Working Group [11,12].

**Figure 2:**
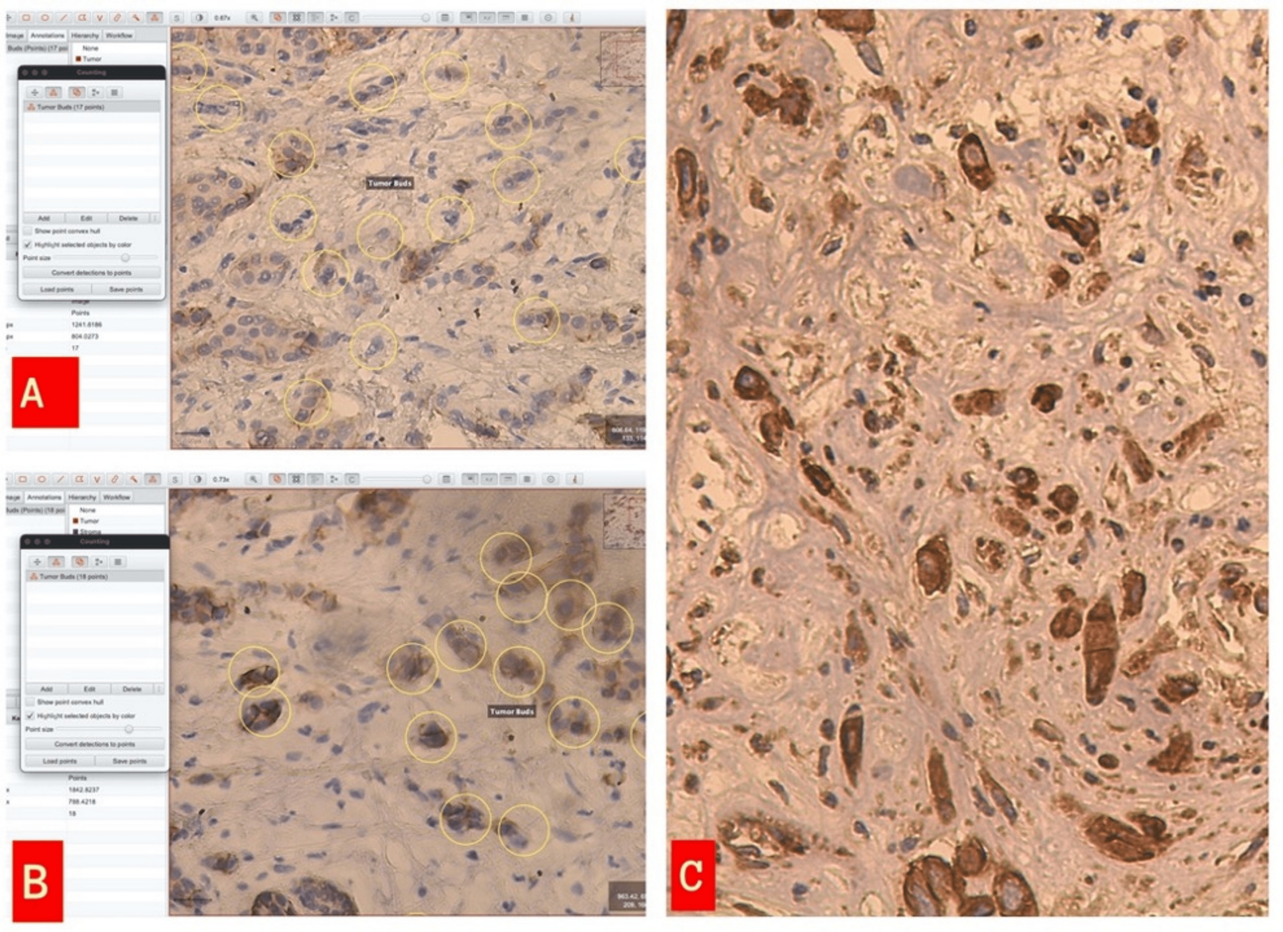
(A) E-cadherin low expression (10%) in tumor buds (immunohistochemistry (IHC), 200× Qupath Program image). (B) E-cadherin moderate expression (30%) in tumor buds (Qupath Program image). (C) E-cadherin high expression (90%) in tumor buds (IHC, 200×).

Immunohistochemical analysis, a technique used to visualize the presence, abundance, and localization of specific proteins in cells, was performed using the Leica primary antibody vimentin (Clone: V9 Lot: 71990) and β-catenin (Clone: 17C2 Lot: 64174) to evaluate the expression of E-cadherin (Clone: 36B5 Lot: 69423). These markers were chosen because they are commonly associated with the EMT process. The Novolink Polimer detection system was used as a visualization system. The expression of immunohistochemical markers was evaluated in tumor buds (Figure 2).

**Figure 3:**
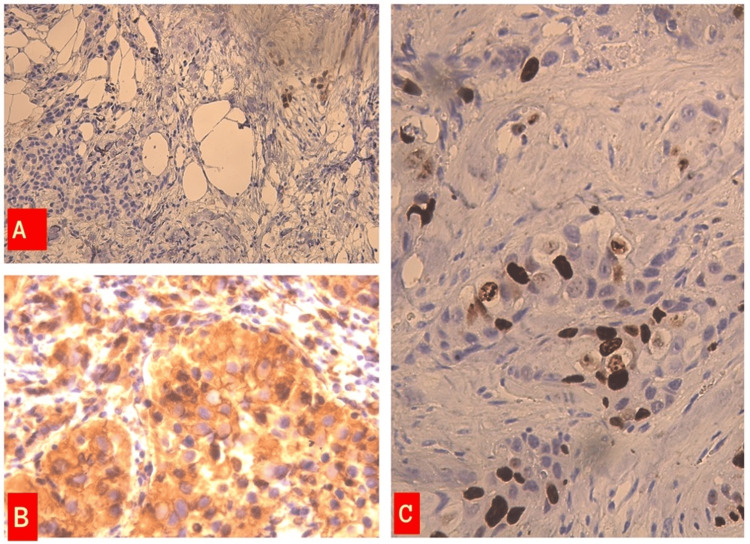
(A) Beta-catenin low expression in the primary tumor area (8%) (immunohistochemistry, 200×). (B) Vimentin expression in the primary tumor area (IHC 300×). (C) Beta-catenin expression in the primary tumor area (IHC, 400×).

The expression of immunohistochemical markers was evaluated in the primary focus of the tumor using the same algorithm (Figure 3).

## Results

Table 1 presents the characteristics of the study participants. The average age of the patients included in the study was 44 years.

**Table 1:** Characteristics of the study group.

Characteristic	Total (n = 137)
Age (years)	
Mean (range)	44 (35–70)
Tumor stage (T)	
T1	20%
T2	30%
T3	25%
T4	25%
Nodal involvement (N)	
N0 (no involvement)	40%
N1 (1–3 nodes)	35%
N2 (4–9 nodes)	15%
N3 (10+ nodes)	10%
Metastasis (M)	
M0 (no metastasis)	80%
M1 (metastasis)	20%
Tumor grade	
Grade 1	15%
Grade 2	45%
Grade 3	40%
Lymphovascular invasion	
Positive	60%
Negative	40%
Tumor buds	
Mean (range)	14 (5–20)
Tumor-infiltrating lymphocytes	
Mean percentage (range)	25% (10%–50%)

Correlation analysis

**Figure 4:**
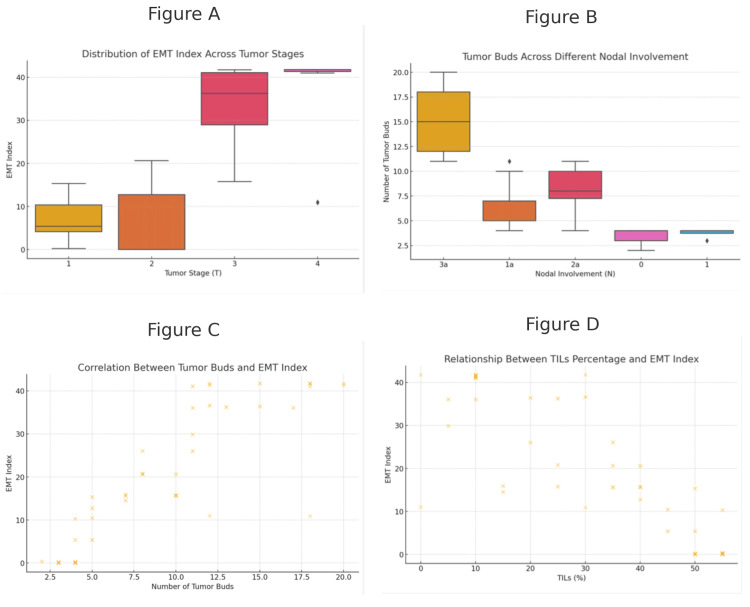
Combined visual analysis of EMT index, tumor buds, and TILs across various tumor characteristics. A: The distribution of the EMT index across different tumor stages (T1, T2, T3, T4) using a boxplot, highlighting variations as the tumor stage progresses. B: The distribution of tumor buds across different levels of nodal involvement (N) through a boxplot, emphasizing the relationship between nodal involvement and the number of tumor buds. C: A scatter plot correlating the number of tumor buds with the EMT index, revealing a trend in their association. D: The relationship between TIL percentage and the EMT index in a scatter plot, showing how TILs might influence or correlate with EMT index levels. This figure provides a comprehensive overview of key markers and their relationships within the tumor microenvironment. EMT: epithelial-mesenchymal transition; TIL: tumor-infiltrating lymphocyte

The analysis showed statistically significant positive correlations between the EMT-related markers in different tumor regions of TNBC. Strong positive correlations of vimentin levels were found between primary tumor sites, lymph node metastases, and the number of tumor buds (Pearson r = 0.90, r = 0.90 to 0.92; p < 0.001, p < 0.001), supporting its general use as a mesenchymal marker in the EMT process throughout all anatomical locations analyzed. By contrast, there were strong negative correlations of E-cadherin with vimentin in all tumor regions (Pearson r =−0.81, r= −0.81 to −0.89; p < 0.001, p < 0.001), confirming its essential role as a key epithelial marker and concomitant downregulation during dysfunctioning EMT efforts. In contrast, beta-catenin displayed less consistent correlations with other markers, likely reflecting variations in the regulation and functions of EMT that might depend on additional molecular pathways or stromal interactions (Figure 4).

Regression analysis

The effect of vimentin, the only independent variable in our multiple linear regression model, was significant in predicting the EMT index (β = 1.0039, p < 0.001, p < 0.001). E-cadherin (β = −0.0159, p < 0.001, p < 0.001) and beta-catenin (β = +0.0105, p < 1×10^−6^]. An R-squared value of 1.000 in the model reveals that nearly all variability found in the EMT index is explained by independent variables now used as predictors. However, many of these predictors had high variance inflation factor (VIF) values (>3), which may have inflated this reported correlation.

Tumor buds and the epithelial-mesenchymal transition index

**Figure 5:**
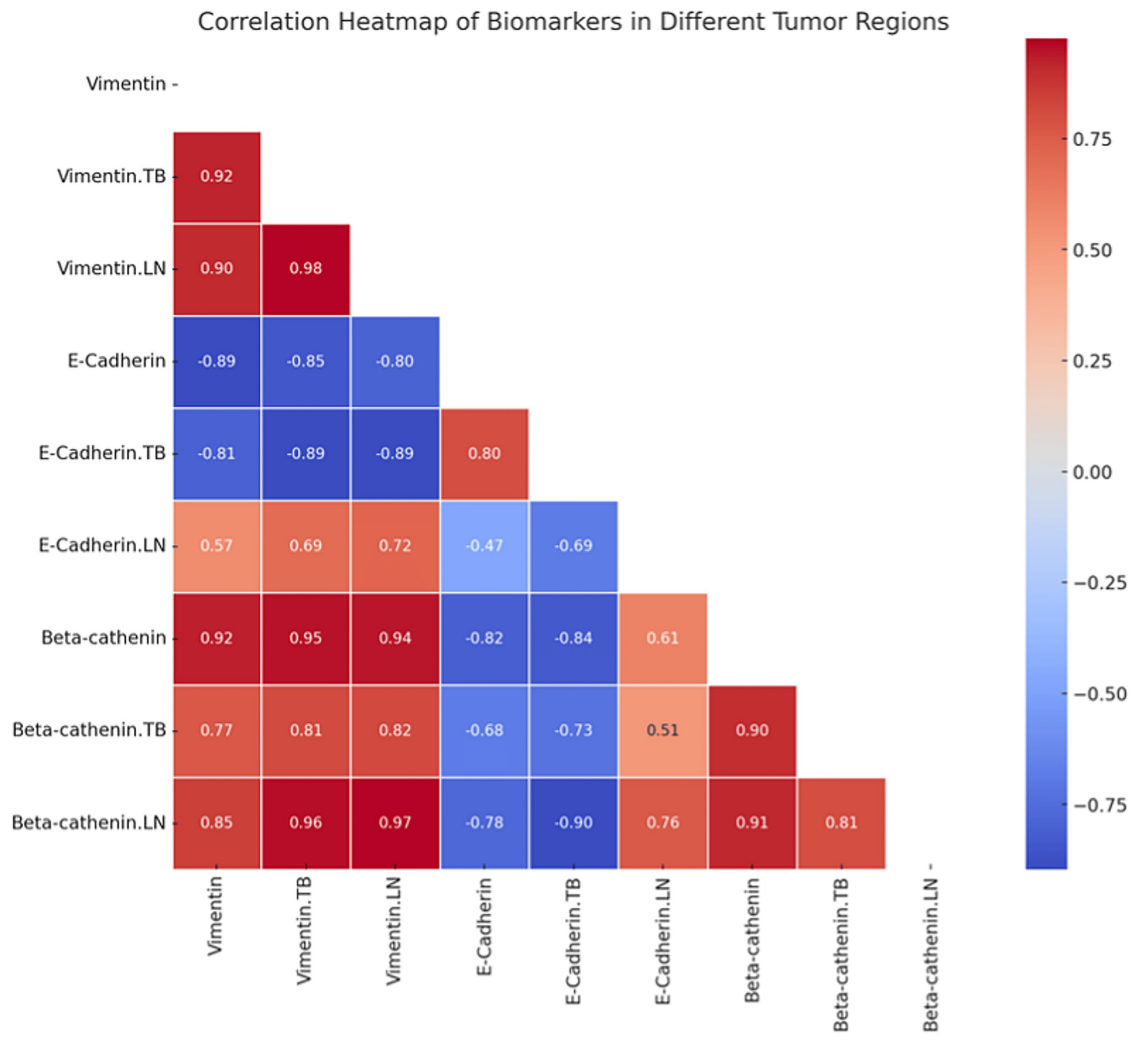
Correlation matrix heatmap. The heatmap visually represents the correlations between various EMT markers, tumor buds, and TILs. The strength and direction of the correlations are color-coded, making it easy to interpret the relationships. EMT: epithelial-mesenchymal transition; TIL: tumor-infiltrating lymphocyte

There was a significant positive correlation between tumor buds and the EMT index (Pearson r = 0.91, p < 0.001). This relationship supports the concept that a high number of tumor buds are associated with an elevated EMT index, and suggests their usefulness as objective biomarkers for cancer progression/advancement in relation to EMT (Figure 5).

Tumor-infiltrating lymphocytes and the epithelial-mesenchymal transition index

TILs demonstrated a strong negative association with the EMT index (p < 0.0001, r = −0.9). The inverse relationship observed between TILs and the EMT index implies that as the levels of TILs increase, this results in a concomitant decrease in tumor aggressiveness driven by EMT.

## Discussion

Strong positive correlations of vimentin in multiple tumor regions validate its utility as a well-established mesenchymal marker in response to the EMT course, confirming that it may serve as an ideal predictor for activation or completion stage in an oncogenic context in TNBC [13]. The negative relationship between E-cadherin and vimentin is consistent with the prototypical concept of EMT, in which an epithelial trait (E-cadherin) decreases or increases as a mesenchymal one does [14-16]. This interplay is critical for explaining the aggressive phenotype of TNBC and underscores their role as prognostic or genetic features to target therapeutically. The strong positive association between tumor buds and the EMT index supports using the quantification of tumor buds as a proxy for assessing EMT in TNBC.

This further confirms the central place of vimentin within the EMT process and proposes it as a strong predictive marker for TNBC survival with high prognostic capability using regression models [17,18]. The contribution of E-cadherin and beta-catenin to the EMT index was higher, indicating their importance in the progression phase in TNBC. Nevertheless, given high VIF values indicate multicollinearity of the predictors, individual contributions must be read with care. One potential way of addressing some of these problems is by summarizing the complex interplay among different immune markers into more stable and interpretable metrics through dimensionality reduction techniques, e.g., principal component analysis.

Tumor buds as a biomarker

A significant positive association of tumor buds with the EMT index indicates the potential importance of using tumor buds for evaluating cancer aggressiveness in TNBC. Detection of tumor buds and their count may indicate a far down EMT track, which is informative in prognosis as well as reveals novel aspects for intervention.

Tumor-infiltrating lymphocyte and tumor suppression

The inverse relationship between the TIL score and the EMT index supports the potential role of the host immune response to counteract EMT, a critical event in tumor progression. These findings suggest that increased TILs may represent an effective immune perturbation counteracting the mesenchymal phenotype and perhaps crystallize in novel therapeutic manipulations designed to augment immune-mediated repression of aggressive tumor behavior.

Future investigations should replicate these findings in larger cohorts and across the TNBC subtypes. Another possibility is focusing on combined therapeutic strategies to target multiple EMT-related pathways in parallel, which may provide additional clues if monotherapy proves ineffective. The development of these immune-modulating therapies along with strategies to augment TIL functionality against EMT should be the focus of future studies.

## Conclusions

In this study, we conducted a comprehensive examination of the interactions between EMT-related biomarkers in TNBC, revealing their coordinated roles in disease progression and metastasis. By addressing the statistical challenges of multicollinearity through PCA, we enhanced the predictive accuracy and clinical relevance of these biomarkers. This approach not only made predictive models more robust but also uncovered new dimensions for designing mechanisms of targeted therapeutics.

Our findings emphasize the importance of a holistic strategy in targeting EMT in TNBC. Rather than focusing on individual biomarkers, the research underscores the significance of their collective regulation during the EMT process. An integrated perspective like this may lead to the identification of synergistic interactions crucial for understanding tumor phenotype and improving therapeutic strategies. Ultimately, these insights expand our understanding of TNBC’s molecular underpinnings and suggest that EMT markers have significant potential as prognostic indicators and therapeutic targets. Future research should explore the combined impact of these markers, considering the potential benefits of multi-targeted therapies that address the complex EMT environment active in TNBC.
